# Spontaneously Disappearing Calcifications in the Breast: A Rare Instance Where a Decrease in Size on Mammogram Is Not Good

**DOI:** 10.7759/cureus.8753

**Published:** 2020-06-21

**Authors:** Quan D Nguyen, Nga T Nguyen, Linden Dixon, Flavia E Posleman Monetto, Angelica S Robinson

**Affiliations:** 1 Radiology, University of Texas Medical Branch, Galveston, USA

**Keywords:** disappearing calcifications, screening mammogram, spontaneously resolving calcifications, breast cancer, invasive ductal carcinoma, spontaneously decreasing calcifications

## Abstract

Spontaneously resolving breast calcification on mammography is a rare radiologic finding. This phenomenon is defined by a decrease in number and/or prominence of breast calcifications on mammogram when compared to prior imaging. The significance of resolving breast calcifications remains unclear, but they have been reported in cases of malignancy. In current literature, patients whose imaging illustrated a decrease in calcifications usually had other concomitant breast complaints. We are presenting a case of invasive ductal carcinoma, in which the patient was asymptomatic on physical examination. Spontaneously resolving breast calcification and lymphadenopathy were the only abnormal findings on screening mammogram.

## Introduction

Breast cancer is the most frequently diagnosed cancer type and the second leading cause of cancer-related deaths among female patients, accounting for 23% of total cancer cases and 14% of cancer-related deaths [1]. Despite this escalation, easy accessibility to high-quality screening modalities and advanced treatments has caused the annual mortality rate from breast cancer to decrease significantly at 1.9% per year from 2008 to 2012 [1]. Physical examination and mammography are the most common screening methods for breast pathology. Compared to physical examination, screening mammography detects breast cancers that are smaller, more likely to be treated with breast conservation surgery, and less likely to have metastasized and to receive adjunct chemotherapy [2]. As a result, it is crucial for radiologists to characterize abnormalities on mammography prior to making a final recommendation for management, which permits accurate diagnoses and prompt treatments for breast cancer patients at early stages.

The two main mammographic findings suggestive of a breast cancer are masses and calcifications. The presence of a suspicious mass or calcifications is one of the first signs of breast cancer. Masses are characterized by their shapes and margins, while calcifications are specified by their morphologies and distributions. Approximately one-fifth of invasive cancer cases diagnosed by mammography directly result from calcifications on imaging [3]. Therefore, differentiating the varieties of calcifications is crucial in assessing the likelihood of malignancy.

Breast findings that decrease in size are almost always nonmalignant; however, decreasing calcifications is not a benign finding on mammogram. Unlike small tumors whose sizes have been recognized to be directly correlated with higher chances of cancer survival, spontaneous decreases of calcifications are not necessarily indicative of better clinical outcomes. This unusual finding is rare. In current literature, disappearing calcifications have been scarcely reported in fewer than 10 studies. It has been demonstrated that a decrease in or complete resolution of breast calcifications is most concerning when it is associated with an extra breast mass, architectural distortion, or increased density [4,5]. In this paper, we present a rare case of spontaneously resolving breast calcifications on screening mammography whose biopsy was later found to be invasive ductal carcinoma with axillary lymph node metastasis. The patient was asymptomatic and had no new breast masses on physical examination. Our case illustrates that spontaneously resolving calcifications may be the only concerning sign of malignancy on imaging. Highlights of imaging features and a review of current literature are presented.

## Case presentation

In December 2018, a 54-year-old woman presented herself for a well woman exam. Her past medical history was significant for right breast abnormal calcifications on a previous mammogram performed in 2016. The patient stated a biopsy was recommended but she could not afford the procedure. She also had a breast fibroadenoma of the right breast at age 30 years, which was removed. The patient had no known family history of breast cancer. Her past surgical history included three cesarean sections and a bilateral tubal ligation. She self-reported as a nonsmoker. On physical examination of both the right and left breasts, no mass, tenderness, or swollen lymph node was appreciated. The patient presented for a routine screening mammogram.

**Figure 1:**
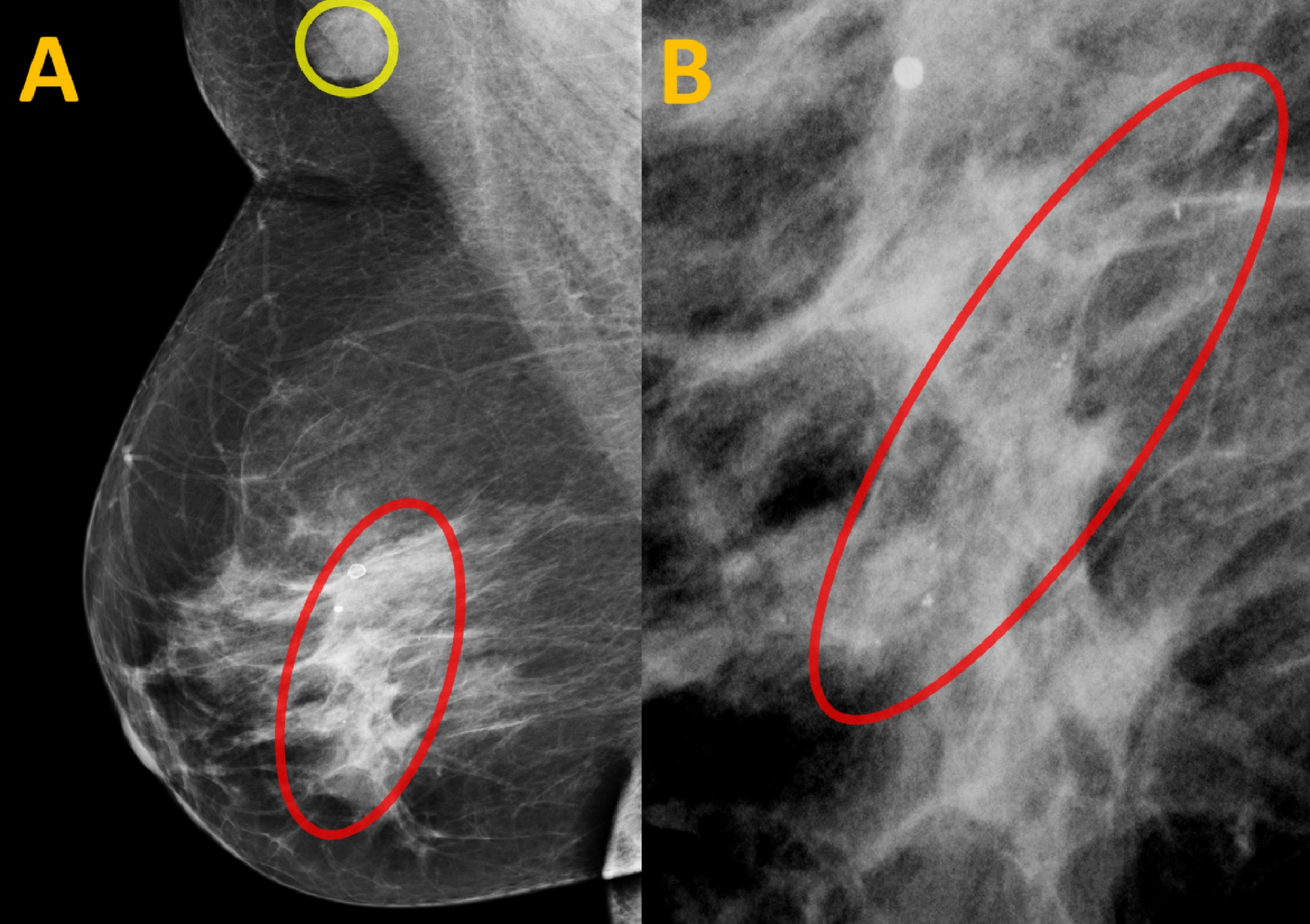
Current Screening Mammogram of the Right Breast in the Mediolateral Oblique View (A) This mammogram image of the right breast shows amorphous calcifications in a linear distribution at 8 o’clock at a distance of 6 cm from the right nipple (red circle). An enlarged axillary lymph node (yellow circle) is also present. (B) A close-up view of the right breast mammography shows the same amorphous calcifications, spanning 26 mm (red circle).

Her repeat mammography revealed right breast amorphous calcifications in a linear distribution spanning 26 mm at 8 o’clock at a distance of 6 cm from the nipple (Figure 1). A morphologically abnormal lymph node measuring 15 mm was also noted in the right axilla (Figure 1A).

Because of these abnormal findings, her lesions on mammography were classified according to the Breast Imaging, Reporting and Data System (BI-RADS) as BI-RADS category 4 suspicion for malignancy. In the left breast, there was no evidence of suspicious masses, calcifications, or other abnormal findings. In consideration of her age, abnormal mammogram in the past, current amorphous calcifications, and abnormal lymphadenopathy, primary breast cancer was raised as a concern. Therefore, the mammogram in 2016 was subsequently obtained from an outside hospital for comparison.

**Figure 2:**
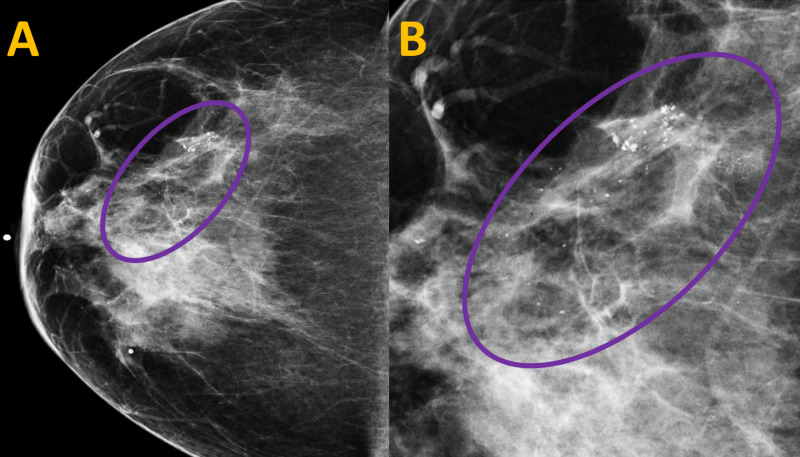
Previous Screening Mammogram of the Right Breast in the Craniocaudal View (A) A mammogram of the right breast performed in 2016 shows amorphous calcifications in a linear distribution at 8 o’clock at a distance of 6 cm from the right nipple (purple circle). (B) A close-up view of the mammogram image demonstrates the same amorphous calcifications, spanning 49 mm (purple circle).

The previous mammogram of the right breast revealed amorphous calcifications in a linear distribution spanning 49 mm at 8 o’clock at a distance of 6 cm from the nipple which was at the same location as the current calcifications (Figure 2).

**Figure 3:**
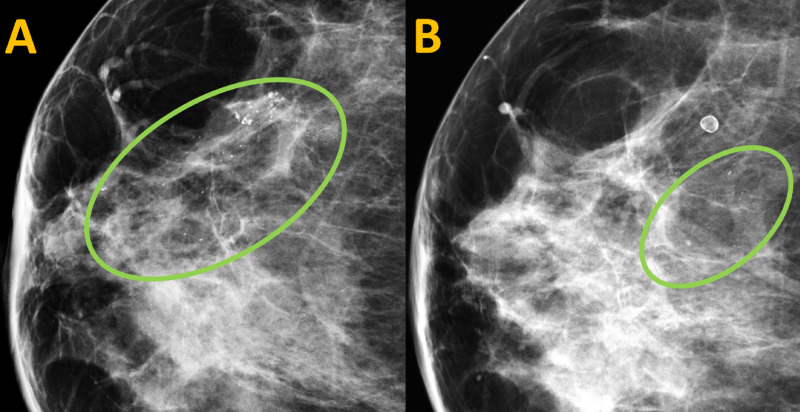
Comparison Images of the Current and Previous Mammograms of the Right Breast in the Craniocaudal View (A) Previous mammogram of the right breast. (B) Current mammogram of the right breast. Compared to the previous mammogram, amorphous calcifications (green circles) in a linear distribution spanning 49 mm are noted to have decreased to 26 mm on the current mammogram. These comparison images present an uncommon mammographic finding of resolving calcifications.

Compared to her previous mammogram, the linearly distributed calcifications were noted to have decreased from 49 mm to 26 mm in anterior posterior dimension on the current mammogram (Figure 3).

**Figure 4:**
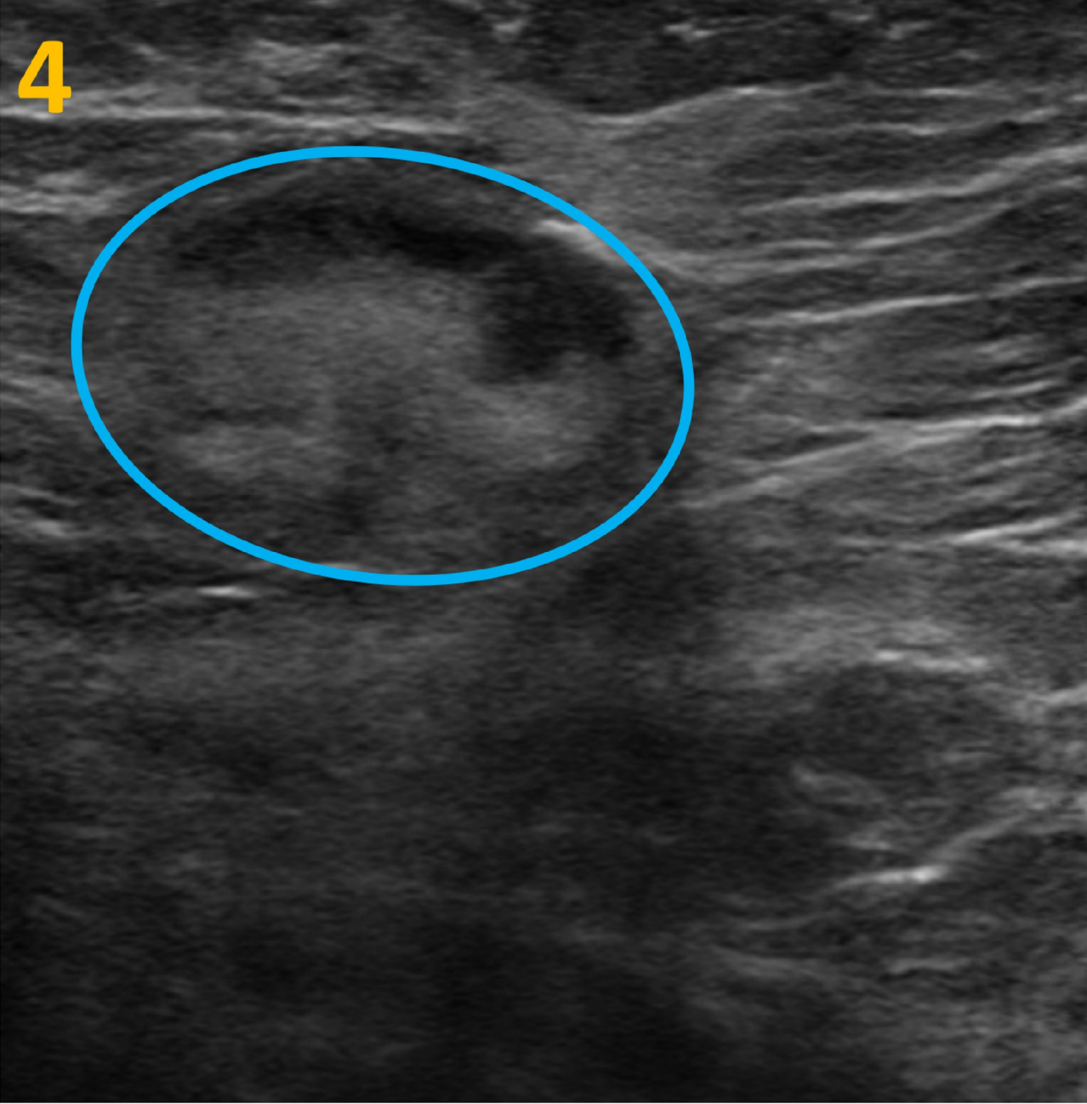
Targeted Ultrasound of the Right Axilla This ultrasound image demonstrates a morphologically abnormal lymph node measuring 15 mm with eccentric cortical thickening in the right axilla (blue circle).

These findings presented an uncommon mammographic finding of spontaneously resolving calcifications. Because of the unusual behavior of the calcifications, further ultrasound imaging of the right breast and right axilla was recommended. Ultrasound confirmed a morphologically abnormal lymph node measuring 15 mm with eccentric cortical thickening in the right axilla (Figure 4). However, no sonographic abnormality was identified at the site of the abnormal mammographic calcifications.

The temporal change of the breast calcifications and abnormal lymphadenopathy raised the concern for breast cancer. As a result, biopsies of the calcifications and axillary lymph node were recommended. She agreed to the biopsy during the current visit and received financial assistance to proceed with diagnostic interventions, including a stereotactic core biopsy of the amorphous calcifications and an ultrasound-guided biopsy of the morphologically abnormal axillary lymph node. Histopathological results of the biopsies showed moderately differentiated invasive ductal carcinoma of the right breast with metastasis to the right axillary lymph node. Specifically, the invasive ductal carcinoma was found to be estrogen receptor negative, progesterone receptor negative, and Her-2-neu positive, with a Ki-67 index of 50%. The patient was informed of the results and referred to a breast surgeon for further management.

## Discussion

Mammographic calcifications were first described in 1913. Since then, it has become one of the most common mammography-based indicators of almost one-third of breast cancer cases in women [3]. Based on their sizes, breast calcifications can be differentiated into coarse (larger than 1 mm in diameter), intermediate (0.5-1 mm in diameter), and fine (smaller than 0.5 mm in diameter) types [6]. Typically, benign versus malignant calcifications are classified not only by sizes but also by their morphologies and distributions. Benign calcifications, which are located in skin or around arterial walls, are often coarse, smooth, rim-like, and oval. Meanwhile, features such as pleomorphism, linear and branching forms, segmental distribution within a lobe of the breast, and interval changes are highly suspicious for malignancy [7].

Generally, findings that are new or increase in size compared to prior mammograms are concerning for malignancy. Conversely, when a finding is stable or decreases in size on a mammogram, it is often considered benign. As a result, the focus of radiologists is usually findings that are new or enlarge while resolution of an abnormal finding on mammogram is deemed insignificant. By definition, spontaneous disappearance of calcifications is not dystrophic. It is not caused by trauma, surgery, irradiation, or chemotherapy [8]. This finding is unusual and has been associated with breast carcinoma. Overlooking spontaneously resolving or disappearing breast calcifications can lead to a delay in cancer diagnosis [4,5,8-10].

The association between this rare finding and the likelihood of carcinoma, however, remains controversial in extant literature. In the first case report in 1988, six out of eleven cases of spontaneously resolving calcifications raised suspicion of malignancy on mammography [9]. The characterization of these lesions was based on the classification of Sickles [11]. However, it was unclear if breast cancer was eventually confirmed with tissue biopsy in any of these cases. Parker et al. reported that only one out of 20 foci of resolving calcifications was minimally suspected of an increased risk of cancer, based on imaging finding alone and without histopathological evidence [10]. Seymour et al. showed that over one-third of the patients with resolving indeterminate calcifications later developed cancer [5]. In this study, the characteristics of indeterminate calcifications include a clustered distribution, a round or cluster shape, and a granular or round form [5]. However, the decrease in calcifications was not the only temporal change in these cases. In particular, all the women in this group had additional imaging abnormalities and 75% of the patients reported newly developed breast masses, which altogether already put them in a higher risk classification. More recently, a case detailed a diagnosis of invasive ductal carcinoma after a complete disappearance of calcifications was noticed on imaging [4]. However, this patient also presented with a new dense and irregular mass at the location of the previously documented calcifications. In the case we are presenting, the patient was asymptomatic on physical examination. Her only abnormal findings were spontaneously resolving calcifications on mammography and a swollen lymph node that were later shown to be associated with invasive carcinoma on tissue biopsy.

Although calcifications are one of the most common findings in breast imaging, the mechanisms of its formation and resolution remain unclear. Calcifications are mainly composed of calcium oxalate and calcium phosphate. Because calcium oxalate is produced by apocrine cells in the breast, it has been suggested that calcifications result from cellular hyperactivity in the lobulo-ductal complex or a product of necrotic cellular degeneration [12]. Exposure to high levels of oxalate has also been shown to precipitate cellular and genetic changes [13]. Compared to calcium oxalate, calcium phosphate is more commonly found in malignant lesions in an unknown mechanism [14]. Similarly, disappearance of breast calcification also requires further research. This unusual phenomenon is thought to be caused by the dissolution of calcifications, rather than excretion through the ductal system. Local factors, including pH and calcium and phosphate ion concentrations, could also play a role [10].

Because mammography alone does not clearly differentiate benign from malignant lesions, BI-RADS classifications are helpful in determining the next course of action. Particularly, breast lesions with the BI-RADS 4 and above should prompt further intervention because of the increased likelihood of cancer [15]. Although spontaneously disappearing calcifications have not been well characterized in the BI-RADS system, indeterminate coarse heterogeneous or amorphous calcifications alone would be assessed as BI-RADS 4. In this case, considering the positive mammography in the past, along with the amorphous morphology, linear distribution, and unusually disappearing pattern of calcifications on the current imaging, we recommended core needle biopsies of the right breast calcifications and right axillary lymph node. The biopsies confirmed right breast invasive ductal carcinoma with axillary lymph node metastasis.

## Conclusions

We have detailed a case in which disappearing calcifications on mammography are associated with invasive ductal carcinoma. As this rare finding raises a suspicion of malignancy, further evaluation with more imaging and possible tissue biopsy should be recommended to patients. In our case, additional diagnostic mammogram and ultrasound revealed no other abnormalities in the breast, but axillary lymphadenopathy was identified. Our case report emphasizes the importance of recognizing decreasing calcifications on mammograms and pursuing further evaluation with additional diagnostic mammogram and ultrasound workup. Stereotactic-guided biopsy of the area should be performed to evaluate for malignancy. Since the exact mechanism of resolving calcifications remains unknown, future research focused on the pathogenesis of this rare phenomenon can shed light on its significance and relationship with other disease processes.
